# Neuromorphic Devices for Bionic Sensing and Perception

**DOI:** 10.3389/fnins.2021.690950

**Published:** 2021-06-29

**Authors:** Mingyue Zeng, Yongli He, Chenxi Zhang, Qing Wan

**Affiliations:** School of Electronic Science & Engineering, Nanjing University, Nanjing, China

**Keywords:** neuromorphic devices, artificial neural systems, artificial intelligence, bionic sensing and perception, neuromorphic engineering

## Abstract

Neuromorphic devices that can emulate the bionic sensory and perceptual functions of neural systems have great applications in personal healthcare monitoring, neuro-prosthetics, and human–machine interfaces. In order to realize bionic sensing and perception, it’s crucial to prepare neuromorphic devices with the function of perceiving environment in real-time. Up to now, lots of efforts have been made in the incorporation of the bio-inspired sensing and neuromorphic engineering in the booming artificial intelligence industry. In this review, we first introduce neuromorphic devices based on diverse materials and mechanisms. Then we summarize the progress made in the emulation of biological sensing and perception systems. Finally, the challenges and opportunities in these fields are also discussed.

## Introduction

In addition to the achievements of artificial intelligence (AI), the engineering community has been trying to learn and imitate biological neural systems from almost all aspects, such as neural networks and robots, in order to seek artificial general intelligence (AGI). Biological neural systems have the characteristics of in-memory computing structure, large-scale parallel processing and event driven operation (Kuzum et al., 2013; Indiveri and Liu, 2015; Furber, 2016). Such characteristics allow us to perceive and react precisely when confronting to events of the real world in robust, fault tolerant and energy efficient modes (Merolla et al., 2014). Because of these, biological neural systems are more efficient and faster than any hardware/software computing platforms can accomplish for the given scale (Lohman, 1989; Picard et al., 2001). As the solution for efficient processing of enormous dataset sizes, electrical implementation of neural systems has been an important approach for external information acquisition (e.g., sensor), and more significantly, the external information processing and execution through cognitive processes (e.g., neuromorphic computing systems) (Delbruck et al., 2014). Artificial synapses can control information flow, data processing and memory function by modulating the synaptic weight (Voglis and Tavernarakis, 2006; Gkoupidenis et al., 2015). Hardware implementation of synaptic/neuronal functions represents a new-concept paradigm (Park et al., 2020).

**FIGURE 1:**
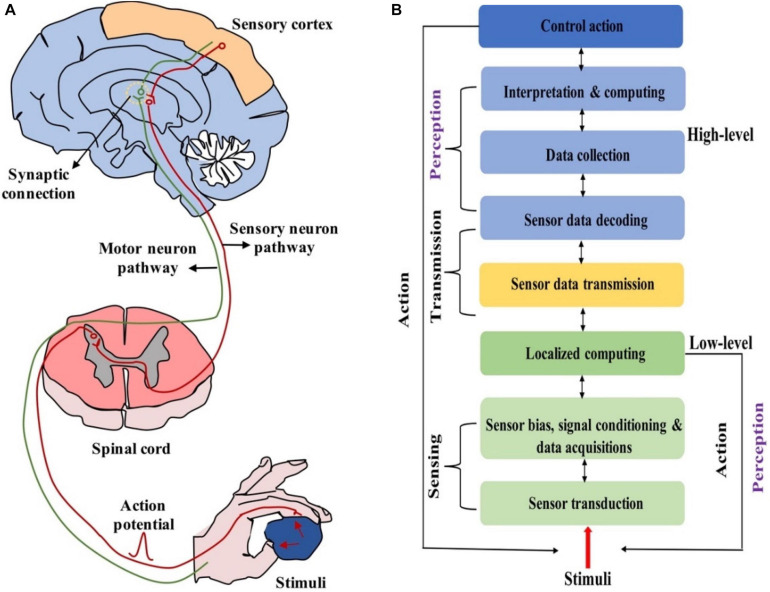
**(A)** Biologically neural systems for sensing and perception. **(B)** Hierarchical functional framework of information processing in neuromorphic neural system (adapted from Wang et al., 2020).

The biological neural systems play the leading role in the regulation of physiological functions of vertebrates’ body. Such systems are mainly divided into two parts: central neural system (CNS,

i.e., the brain and spinal cord) and peripheral neural system (PNS, i.e., the sensory and motor nerves) (Lee and Lee, 2019). The CNS conducts high-level activities such as learning, memorizing and planning, and dominates the activities of the body in response to the information received from PNS. The PNS perceives and reacts to stimuli, e.g., light, sound, chemicals and pressure, and transmits this information between CNS and other parts of the body (Anderson, 2010). It’s worth noting that PNS can perform some low-level activities such as reflex and muscle activation directly (Wang et al., 2020). In other words, such sensory signals don’t need to be sent to the brain, and a perceptual decision is made immediately once the sensory signal reaches the spinal cord (Figure 1A). Compared with the brain, PNS processes low-level perceptual activities in a decentralized and localized manner. Localized processing cannot only quickly respond to external stimuli to maintain survival, but also reduce the computational burden of the brain (He K. et al., 2020; Tuchman et al., 2020; Figure 1B). Due to such merits, localized processing has been employed in interactive robotics (Groothuis et al., 2018; Wang et al., 2020).

As we know, neuromorphic engineering aims to build bio-inspired cognitive systems to emulate biological neural sensing and processing capabilities; in the meantime, neuromorphic engineering derivates synaptic devices (Field, 1994; Cohen et al., 1997; Kandel, 2001). When performing tasks like pattern classification and feature extraction, artificial neural network based on synaptic devices can effectively implement machine learning algorithms (Lumpkin and Caterina, 2007; Dahiya et al., 2010; Abraira and Ginty, 2013). Inspired by the mentioned above, the incorporation of bio-inspired sensing and neuromorphic engineering technologies for emulating the functions of CNS and PNS may open up a new era for artificial intelligence. In pursuit of mimicking the CNSs and PNSs, two methods have been adopted: the development of synaptic devices with capabilities of information processing and detection of stimuli such as light, sound, chemicals and pressure; the development of artificial synaptic devices integrated with sensing elements (e.g., light, touch) (Park et al., 2020). The approach of device integration has been widely demonstrated for constructing artificial neural systems in recent years, and this method has the potential to be applied on next-generation wearable electronics, robotics, and neuro-prosthetics (Hammock et al., 2013; Wan et al., 2018). For example, artificial neural systems can help to replace damaged neurons, or they can be employed as tools in neuroscience to study sensory/motor neuronal disorders. Furthermore, artificial neural systems can be developed to realize sensory information extraction and analysis, as well as to settle problems in uncertain settings. These systems that able to perceive the environment and react accordingly will cast significant impact on the progress of artificial intelligence.

Here, recent progress in the development of neuromorphic devices for mimicking the bionic sensing and perception functions will be discussed. Firstly, we introduce the properties of the biological synapses and the diverse synaptic characteristics that should be mimicked by neuromorphic devices. Second, we discuss the neuromorphic devices based on various materials and operation mechanisms. Third, bionic sensing and perception for personal healthcare monitoring, robotics and neuro-prosthetics are reviewed. Last, we summarize the review and give a brief outlook. We are looking forward to providing a guideline for the future development of neuromorphic perception systems.

## Biological Properties of Synapse

**FIGURE 2:**
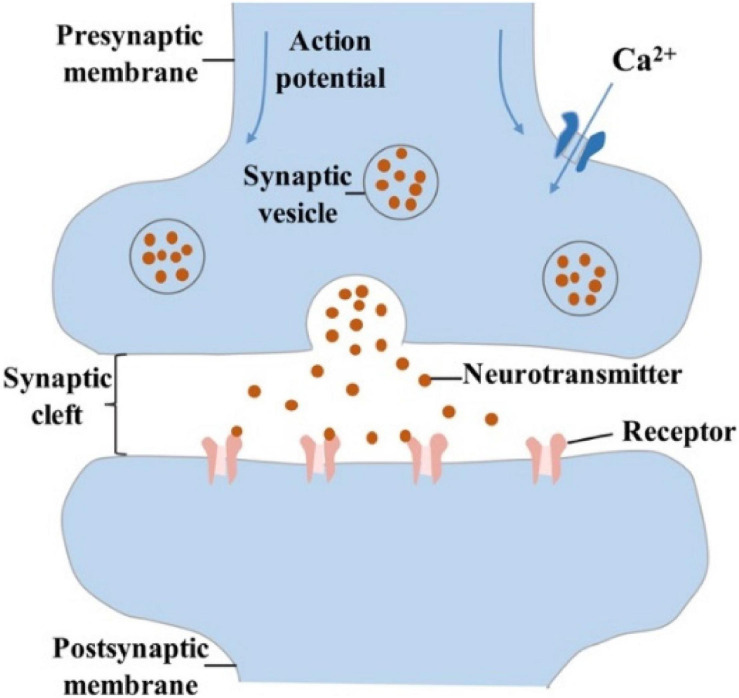
Schematic diagram of chemical synapse, that is composed of presynaptic membrane, synaptic cleft, and postsynaptic membrane.

In the biological nervous system, synapses are divided into electrical synapses and chemical synapses; the former commonly exist in fish and amphibians, while the latter are common in mammals (Schmitt et al., 1976; Connors and Long, 2004; Pereda, 2014; Park et al., 2020). The origin and development of synaptic devices are inspired by the human brain, so only chemical synapses will be discussed. Synapses are composed of three parts: presynaptic membrane, synaptic cleft and postsynaptic membrane (Tsodyks et al., 1998; Pereda, 2014). When an action potential is transmitted to the synaptic corpuscle through axons, the permeability of the presynaptic membrane to Ca^2+^ increases. Then Ca^2+^ in the synaptic cleft enter the synaptic corpuscle, which promotes the close fusion between synaptic vesicle and presynaptic membrane (Mayford et al., 2012). The neurotransmitters in the vesicle are released into the synaptic cleft (Figure 2). Finally, they reach the postsynaptic membrane through diffusion, and immediately bind to the protein receptors on the postsynaptic membrane, causing excitatory postsynaptic potential/current (EPSP/EPSC) or inhibitory postsynaptic potential/current (IPSP/IPSC) (Neher, 1992; Mayford et al., 2012). The spatial sum of EPSC and IPSC (i.e., the sum of postsynaptic potentials that appear at different positions of the neuron) and the time sum of EPSC and IPSC (i.e., the sum of postsynaptic potentials that occur repeatedly at each synapse) are the criterions to judge whether they can trigger action potentials or not (Ferster and Jagadeesh, 1992; Thomson and Deuchars, 1994; Lewis et al., 2011; Pereda, 2014).

The efficiency of information transmission between anterior and posterior neurons is defined as synaptic weight (synaptic strength), and the numerous mechanisms in changing the synaptic weight are collectively called synaptic plasticity (Martin et al., 2000; McGaugh, 2000; Mayford et al., 2012; Liao et al., 2014). Synaptic plasticity is grouped into STP (short-term plasticity) and LTP (long-term plasticity) by the retention time, and both of them have potentiation and depression states. The duration of STP occurs between milliseconds and minutes, which is related to the computational functions of neural network and short-term memory (STM) (Morris, 1999). PPF (paired-pulse facilitation), defined as 100% × A2/A1, where A1 and A2 are the amplitudes of the first and the second postsynaptic current corresponding to two consecutive spikes that divided by a time interval Δt, is a form of STP. PPF participates in neuronal tasks, such as simple learning and information processing (Regehr, 2012; Bornschein et al., 2013). LTP can produce plastic changes that last for a few hours even longer; LTP is related to learning and long-term memory (LTM) (Bear and Malenka, 1994; Linden, 1994; Kullmann and Lamsa, 2007; Ho et al., 2011). Hebb hypothesized that continuous and repeated stimulation from presynaptic neurons to postsynaptic neurons could cause an increase in the efficiency of synaptic transmission (Morris, 1999). The concept of STDP (spike-timing-dependent plasticity) further improves Hebb’s theory, pointing out that the time relationship between presynaptic and postsynaptic spikes can modulate the synaptic weight (Bi and Poo, 1998; Dan and Poo, 2006; Caporale and Dan, 2008). SRDP (frequency-dependent-synaptic plasticity) is another basic learning mechanism for LTP in the brain, i.e., synaptic weight can be modulated by controlling the frequency of presynaptic spikes (Bliss and Lomo, 1973; Bear et al., 1987; Dudek and Bear, 1992; Law and Cooper, 1994). For instance, high-frequency (20–100 Hz) trains of presynaptic spikes will generate LTP, whereas low-frequency (1–5 Hz) trains result in LTD (Bear and Malenka, 1994).

The development of “brain-like” computing is inseparable from the exploration of brain functions by neurologists. Synapses serve as functional connections between neurons, thence synaptic devices are essential for the emulation of the neural functions mentioned above. In recent years, circuits based on lots of silicon transistors have been explored to mimic synaptic functions, proving its possibility in the application of neuromorphic engineering (Ramakrishnan et al., 2011; Bamford et al., 2012). In order to reduce hardware cost and power consumption, in this review, we focus on the realization new-concept neuromorphic devices for bionic sensing and perception applications.

## Neuromorphic Devices Based on Various Materials and Mechanisms

Numerous materials (e.g., electrochemical metallization materials, phase-change materials, ferroelectric materials, ionic/electronic hybrid materials) have been employed to fabricate artificial synapses for constructing neuromorphic network. To mimic bio-synaptic functions, diverse configurations of synaptic devices have been proposed, including: two-terminal (2-T) devices and three-terminal (3-T) devices. 2-T devices have the advantage of small physical size and easy large-scale integration (Jo et al., 2010; Prezioso et al., 2015; Xu et al., 2016a; Lee et al., 2019). 3-T devices can perform signal transmission and self-learning functions simultaneously (Yuan et al., 2010; Wu et al., 2014; Wan et al., 2015; Kim et al., 2016, 2017; Zhu et al., 2016; Gkoupidenis et al., 2017; Dai et al., 2018; Jerry et al., 2018; He Y. et al., 2020). Multi-terminal synaptic devices with multiple presynaptic terminals can be adopted to emulate information processing (Liu Y.H. et al., 2015; Feng et al., 2017; Jiang et al., 2017). In the section, the recent progresses of the synaptic devices will be introduced. More detailed information will be shown in Tables 1, 2.

**TABLE 1:** Summary of 2-T artificial synaptic devices based on various materials and mechanisms.

Mechanism	Active	LTP	STDP	Application	References
Phase change	Nd:AINO	✓	✓	–	Wan et al., 2019
Metallic filament	SiO_*x*_:Ag	–	–	Nociceptor	Yoon et al., 2018
Metallic filament+ Phase change	Lignin	50 s	–	–	Park and Lee, 2017
Ion migration	pMSSQ:Cu^+^	✓	✓	–	Wu et al., 2017
Ion migration	Collagen	–	✓	–	Raeis-Hosseini et al., 2018
Redox reaction	PEDOT:PSS	10^4^s	✓	Face recognition	Wang et al., 2018b
Ferroelectric	P(VDF-TrFE)/PFO	–	–	Image recognition	Tu et al., 2018
Ferroelectric	BiTiO_3_	>10^4^s	✓	–	Ma et al., 2020
Charge trapping	PVPy/AuNPs	✓	–	–	Zhang S.-R. et al., 2019
Oxygen vacancy	WO_*x*_	100 s	✓	–	Lin et al., 2018
Oxygen vacancy	MgO/ZnO	10^4^s	✓	–	Dang et al., 2018
Electron spin	CoFeB/MgO/CoFeB	–	–	Handwritten digital recognition	Zhang et al., 2016

**TABLE 2:** Summary of 3-T artificial synaptic devices based on various materials and mechanisms.

Mechanism	Substrate	Gate insulator	Channel semiconductor	LTP	STDP	Application	References
Ion migration	Glass	Water	IGZO	–	–	–	Wan et al., 2016b
Ion migration	PET	Nanogranular	IGZO	–	–	–	Zhou et al., 2015
Electrochemical reaction	Paper	Chitosan	IZO	–		–	Wu et al., 2014
Electrochemical reaction	PET	Nafion	PEDOT:PSS/PEI	>9 × 10^4^s	–	Pavlovian Learning Image recognition	van de Burgt et al., 2017
Electrochemical reaction	Si	PEO/LiClO4	Graphene	✓	✓	–	Sharbati et al., 2018
Ferroelectricity	PET/PDMS	P(VDF-TrFE)	Pentacene	✓	✓	–	Jang et al., 2019
Charge tunneling and trapping	PET	Al_2_O_3_/PMMA:C_60_	Pentacene	388 s	–	–	Ren et al., 2018
Charge trapping	PI	Al_2_O_3_	IWO	–	–	–	Tiwari et al., 2018

### Electrochemical Metallization Materials

Electrochemical metallization (ECM) memory composed of top electrode/active layer/bottom electrode sandwiched structure is an important resistive switching memory. ECM memory shows the resistive switching based on the formation/rupture of metallic filament, which generates from the oxidation of active electrode (e.g., Ag, Cu), transport of metal cations through active layer, and reduction at noble metal electrode. The filament dissipates may due to spontaneous diffusion, Joule heating or ionization. Diverse electrochemical metallization materials have been used to fabricate ECM memory, including chalcogenide (Hasegawa et al., 2010; Ohno et al., 2011; Zhang et al., 2013), nitride (Yang et al., 2012), amorphous silicon (Zhang et al., 2017), and polymer (Li et al., 2013; Kim and Lee, 2018).

**FIGURE 3:**
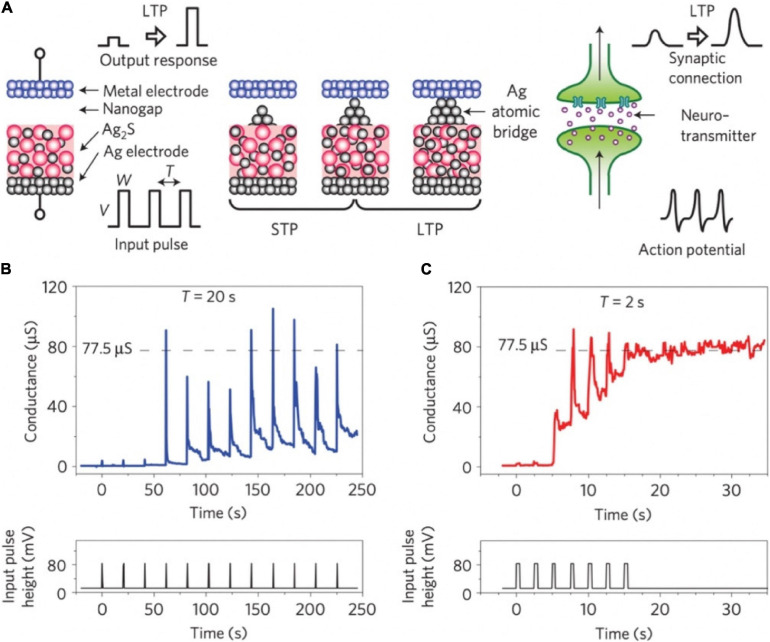
**(A)** Schematic diagram of Ag_2_S inorganic synapse and the formation mechanism for STP and LTP. Right side: The signal transmission of biological synapse. **(B,C)** STP and LTP characteristics of the Ag_2_S inorganic synapse when input pulses were applied with the intervals of 20 and 2 s (adapted from Ohno et al., 2011).

In 2011, an inorganic synapse with the Pt/Ag_2_S/Ag structure was fabricated to emulate the synaptic functions of STP and LTP, see Figure 3A (Ohno et al., 2011). In such synaptic device, the temporal enhancement of conductance occurred before the entire formation of a metallic bridge, and the reduction of the conductance is due to the deformation of instable metallic bridge (Figure 3B). When a robust metallic bridge was formed, a non-volatile conductance enhancement can be obtained, which corresponds to LTP (Figure 3C). In 2017, an ECM memory with Cu/ZnS/Pt structure was fabricated for artificial synapse application, and STP and LTP functions were successfully emulated (Hu et al., 2017). In 2018, single-crystalline SiGe epitaxial random-access memory (epiRAM) exhibited superior spatial/temporal uniformity, and linear weight update based on the confinement of conductive filaments (Ag) into dislocations in SiGe was also observed (Choi et al., 2018).

### Phase-Change Materials

Phase-change materials have been widely explored for memory application (PCM: phase-change memory) because of its scalability, controllable multi-level resistance states and fast read/write speed (Ovshinsky, 1968; Lankhorst et al., 2005; Bichler et al., 2012; Suri et al., 2012; Ambrogio et al., 2016, 2018; Boybat et al., 2018). PCM can be switched from amorphous phase [i.e., high-resistance state (HRS)] to crystalline phase [i.e., low-resistance state (LRS)] by Joule heat (Burr et al., 2016). The application of SET voltage on the PCM causes Joule heating, subsequently raises the material temperature to crystal transition temperature but below melting temperature. As for the RESET operation, material temperature is raised above the melting temperature and then quickly quenched to room temperature; the material solidifies into amorphous phase. By precisely controlling the transition process between amorphous and crystalline states, multi-level intermediate states can be generated to emulate biological synapses (Ielmini et al., 2004; Nakayama et al., 2007; Nirschl et al., 2007). Chalcogenide glass such as Ge_2_Sb_2_Te_5_ (GST) (Ambrogio et al., 2016) and GeTe (Bichler et al., 2012) are commonly applied in phase change memory.

**FIGURE 4:**
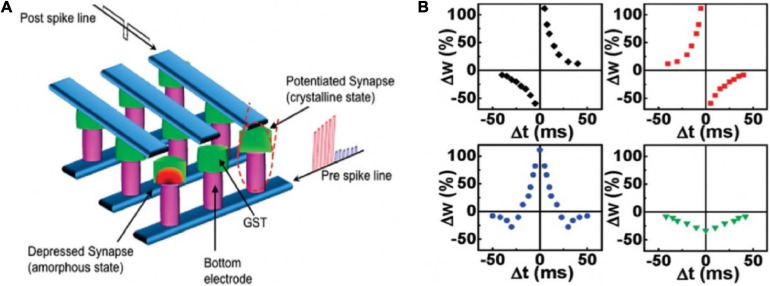
**(A)** Schematic diagram of the crossbar array architecture of the PCM synapses. **(B)** Different forms of STDP learning rules (adapted from Kuzum et al., 2012).

Nanoscale electronic synapses based on GST active layers were fabricated (Figure 4A; Kuzum et al., 2012). Different from biological action potentials, the pre-spike was composed of potentiation (set) pulses with decreasing amplitude and depression (reset) pulses with increasing amplitudes; the post-spike was a continuous pulse with 120 ms duration and with a low negative amplitude pulse of 8 ms in the center. The difference between the pre- and post-spike (V_*pre*_ − V_*post*_) decided the programming voltage applied on the synaptic device at each point. By controlling the time interval in the −50 to 50 ms range, the STDP curve was obtained, which matched with the data measured by Bi and Poo (1998). In addition, the time constant of STDP could be changed by adjusting the amplitude and time spacing of the pulses in the pre-spike. Various STDP characteristics were also demonstrated via swapping the order of depression and potentiation pulses in the pre-spike (Figure 4B). All the synaptic functions displayed by the PCM synapse benefitted from the consecutive transition between intermediate resistance states. Such nanoscale device showed picojoule level energy consumption, making a significant step toward achieving the compactness and energy efficiency traits of brain for future neuromorphic system.

### Ferroelectric Materials

Ferroelectric materials have the properties of high dielectric constant and spontaneous polarization (Chanthbouala et al., 2012; Boyn et al., 2017; Shin et al., 2017; Tu et al., 2018). The polarization states of the ferroelectric materials can be modulated by the applied voltage, so these materials can be used as the active layer in synaptic devices. What’s more, a certain pulse sequence causes the fine polarization states of the ferroelectric material, so multiple conductance states can be obtained in these synaptic devices. The multilevel changes of channel conductance will meet the emulation of various synapse functions. Ferroelectric materials could help synaptic devices improve ON/OFF ratio and linearity of weight updates (Kim and Lee, 2019).

**FIGURE 5:**
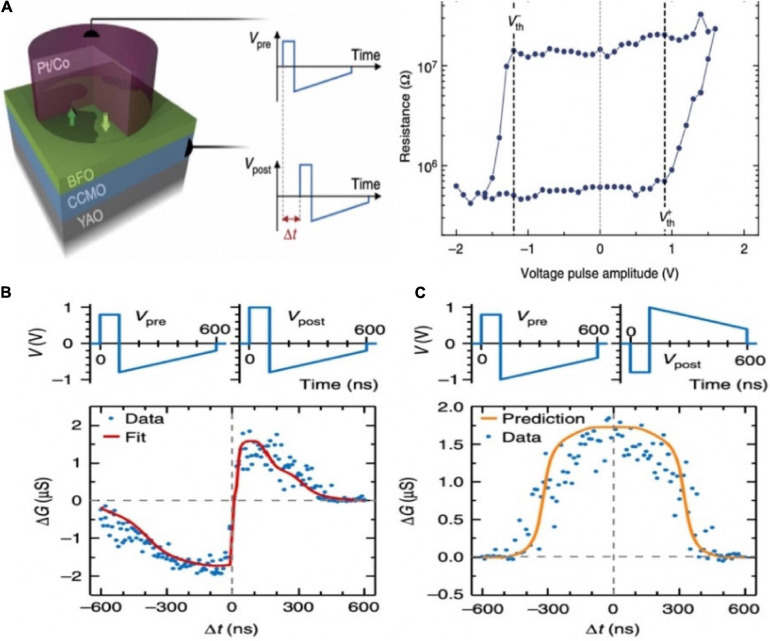
**(A)** Schematic diagram of the ferroelectric memristor. **(B)** The single pulse hysteresis loop of the memristor shows clear voltage thresholds. **(C)** STDP learning curves of different shapes (adapted from Boyn et al., 2017).

An inorganic memristor based on ferroelectric tunnel junctions was constructed to harness the STDP (Boyn et al., 2017). The super-tetragonal BiFeO_3_ (BFO) active layer was sandwiched between Co top and (Ca,Ce)MnO_3_ (CCMO) bottom electrodes, composing the ferroelectric artificial synapse (Figure 5A). As sketched in Figure 5B, well-defined voltage thresholds were shown in the hysteresis loop due to the inherent inhomogeneous polarization switching in the BFO. That made it possible for implementing STDP. By applying various voltage waveforms on the artificial synaptic device, various types of activities between the pre- and post-neuron were realized (Figure 5C).

**FIGURE 6:**
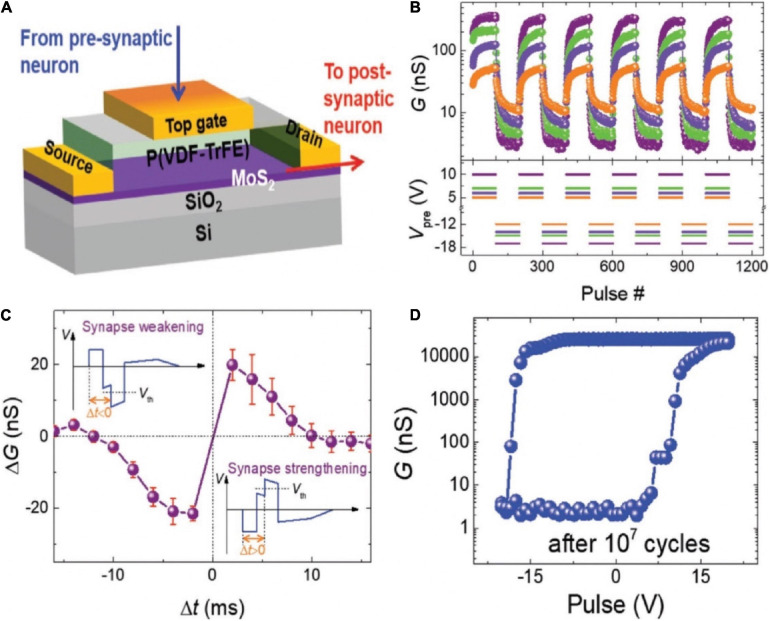
**(A)** Schematic diagram of the organic ferroelectric synaptic device. **(B)** Change of the channel conductance with the different voltage pulse sequences. **(C)** Measurement of STDP in the transistor. **(D)** Change of the channel conductance with various gate voltage pulse amplitude after 10^7^ cycles of total polarization switching (adapted from Tian et al., 2019).

Transistor with ferroelectric material as the gate insulator could be applied as a 3-T memristor device (Yoon et al., 2011; Nishitani et al., 2013). An organic synaptic transistor with ferroelectric poly (vinylidene fluoride/trifluoro ethylene) [P(VDF-TrFE)] was fabricated to mimic synaptic functions (Figure 6A; Tian et al., 2019). The conductance of the device could be modulated alternately between decrement and increment by adjusting the polarity of applied voltages. Thus, the potentiation and depression of synaptic weight could be realized in the device (Figure 6B). STDP was demonstrated by designing the waveforms. The combined waveform temporarily exceeded the threshold voltage, engendering the strengthening (△G > 0) or weakening (△G < 0), depending on the time interval between the pre- and postsynaptic signals (Figure 6C). As shown in Figure 6D, after 10^7^cycles of stimulation of high voltage pulse (± 20 V, 1 ms), the channel conductance could still be modulated to the same highest/lowest states under similar positive voltage and a little increased negative voltage. That organic ferroelectric synaptic transistor has relatively good endurance properties.

Other ferroelectric materials are also utilized as the gate insulator in synaptic devices. For example, Kaneko et al. (2014) used inorganic Pb(Zr,Ti)O_3_ (PZT) ferroelectric film to construct synaptic device for realizing STDP function. Although PZT based ferroelectric transistor exhibits great plasticity in emulating synapses, their inherent Pb content will inevitably bring harm to human beings and environment. HfO_2_ is another feasible candidate for non-volatile memory because of its ferroelectricity and anti-ferroelectricity (Boescke et al., 2011; Park et al., 2015). Recently, ferroelectric material HfZrO_*x*_ was employed in a synaptic transistor, the ferroelectric transistor exhibited excellent plasticity potentiation and depression (Kim and Lee, 2019).

### Ionic/Electronic Hybrid Materials

Ionic/electronic hybrid materials are utilized in field-effect transistors (FETs). Gate dielectric (electrolyte) and channel materials constitute the ionic/electronic hybrid materials. Gate electrolytes have been acted as by diverse materials, such as polyelectrolytes (Yu et al., 2018; He et al., 2019), ion gel (Qian et al., 2016; Xu et al., 2016b), ion liquid (Yang et al., 2017; Yang J.T. et al., 2018), and inorganic oxide (Wan et al., 2013, 2016a). Channel materials can be served as by both inorganic and organic materials, such as IZO (Wan et al., 2016a), IGZO (Zhou et al., 2015), and MoO_3_ (Yang et al., 2017); carbon nanotubes (CNTs) (Kim et al., 2013; Feng et al., 2017); graphene (Tian et al., 2015; Sharbati et al., 2018), MoS_2_ (Jiang et al., 2017; John et al., 2018), and PEDOT:PSS (Gkoupidenis et al., 2015, 2017; van de Burgt et al., 2017). For synaptic FETs, gate electrolytes have good ionic conductivity, in which ions can move randomly, but electrons are not allowed. Synaptic FETs with such gate electrolytes can form electric-double-layer (EDL) at the interface of the channel layer and gate electrolyte. The EDL has a large capacitance value (∼μ*F*cm^–2^ magnitude), so the conductance of the channel layer can be modulated by a small gate voltage, indicating that the FETs could work at a low voltage.

**FIGURE 7:**
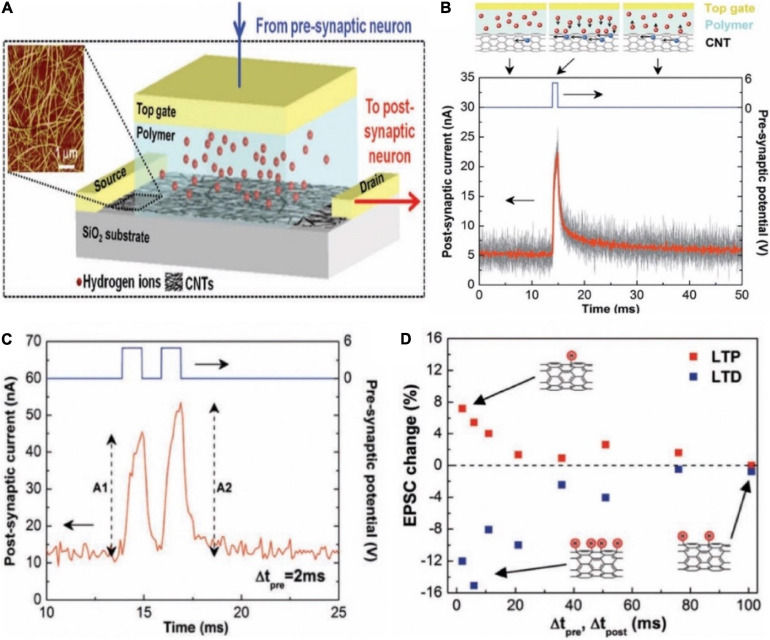
**(A)** The schematic diagram of a carbon nanotube (CNT) synapse, which shows the transistor-like structure of the CNT synapse with a cell containing hydrogen ions in the electrolyte integrated in its gate. Inset is the AFM of a random CNT network. **(B)** EPSC triggered by the pre-synaptic spikes. **(C)** EPSCs triggered by a pair of pre-synaptic spikes. **(D)** The changes of EPSC amplitudes in the synaptic device with Δt_*pre*_ and Δt_*post*_ under the LTP and LTD imitations. Insets illustrate the CNT hydrogenation after the LTD imitation (bottom, left), the dehydrogenation after the LTP imitation (top), and no remarkable change after the LTP or LTD imitations (bottom, right) (adapted from Kim et al., 2013).

In 2013, a carbon nanotube (CNT) synaptic transistor with poly (ethylene glycol) monomethyl ether (PEG) gate dielectric was proposed (Figure 7A; Kim et al., 2013). In the initial state, the hydrogen ions in the polymer were randomly distributed. When a positive voltage pulse was applied on the gate, the hydrogen ions began to move toward the CNTs channel, and an EDL was formed due to the electrostatic coupling effect, causing the channel current increased (Figure 7B). The transistor successfully emulated typical synaptic function PPF (Figure 7C). Other typical synaptic characteristics such as LTP and LTD were also demonstrated (Figure 7D). The CNT synapse had the potential that was integrated in large-scale circuit to emulate the parallel signal processing and learning features of biological neural network.

**FIGURE 8:**
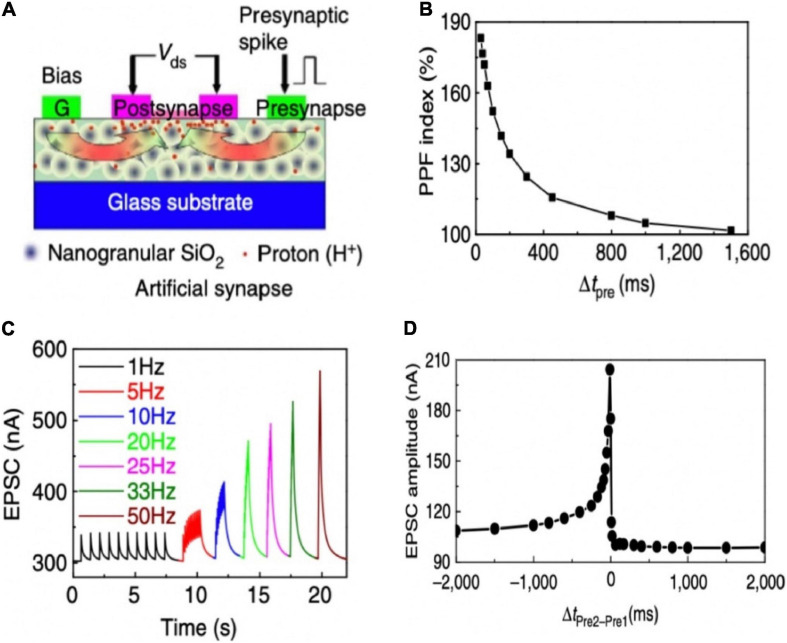
**(A)** Illustration of a laterally coupled IZO synaptic device with two in-plane gates. **(B)** PPF behavior of the IZO synaptic transistor. **(C)** EPSCs triggered by the stimulus trains with different frequencies. **(D)** Dynamic logic testing of the IZO synaptic transistor (adapted from Zhu et al., 2014).

Wan group invented oxide-based electronic/protonic hybrid transistors with in-plane gate configuration. As shown in Figure 8A, an indium-zinc-oxide (IZO) based electronic/protonic hybrid transistor was self-assembled on phosphorus (P)-doped nanogranular SiO_2_ proton conductive films (Zhu et al., 2014). No bottom conductive layer was needed, and the gate voltage could be directly coupled to the IZO channel laterally through only one lateral EDL capacitor. When the in-plane gate was utilized as the presynaptic terminal and the IZO channel with S/D electrodes was acted as the postsynaptic terminal, an artificial synaptic transistor was proposed. The device was employed to imitate a series of short-term plasticity behaviors, including PPF, high-pass filtering behavior and the spatiotemporal correlation dynamic logic (Figures 8B–D). In addition, the laterally coupled synaptic transistor can be easily extended to multiple input gates to realize the function of synaptic interaction. The laterally coupled IZO transistor based on proton conducting electrolyte is of great significance to synaptic electronics and neuromorphic engineering.

**FIGURE 9:**
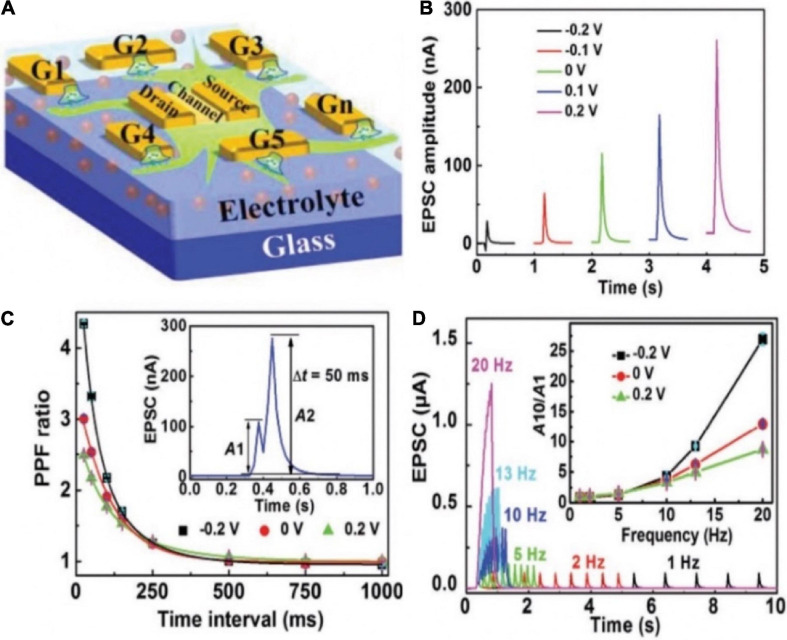
**(A)** Schematic diagram of the multiterminal artificial synaptic device. **(B)** EPSCs triggered by G_1_ with different G_*m*_. **(C)** PPF ratio plotted as the function of time interval between a pair of pulses under different G_*m*_. Inset: EPSCs triggered by a pair of pulses with time interval of 50 ms when there is no G_*m*_ bias. **(D)** EPSCs triggered by spike trains containing 10 spikes with different frequencies when there is no G_*m*_ bias. Inset: EPSC amplitude gain (A_10_/A_1_) changes with presynaptic spike frequency under different G_*m*_ (adapted from He et al., 2019).

Recently, Wan group demonstrated a multiterminal IGZO-based neuro-transistor for dendritic discrimination of different spatiotemporal input modes (Figure 9A; He et al., 2019). Chitosan electrolyte was used as the gate dielectric. The IGZO channel layer, multiple in-plane ITO gate, and ITO source/drain electrodes were deposited by different metal shadow masks. In such neuro-transistor, the synaptic weight can be tuned by the modulatory gate due to the strong lateral electric-double-layer capacitive coupling effect in the electrolyte film. PPF and EPSC behaviors were successfully mimicked in the multiterminal synaptic transistor (Figures 9B,C). In the nervous system, STP contributes to temporal filtering by facilitating or inhibiting the synaptic transmission. Since a larger PPF ratio was obtained with shorter time interval, high-pass temporal filtering could be realized in the transistor (Figure 9D). Various temporal and spatial input patterns of dendrite recognition were also achieved in such multi-terminal neuro-transistor. This kind neuro-transistor can be used as the temporal and spatial information processing unit of basic cortex computing, greatly improving the efficiency of artificial neural network.

## Sensory Applications of Neuromorphic Devices

With the improved understanding of biological sensing process and the development of neuromorphic devices, the application of neuromorphic devices in bionic sensing and perception comes naturally. In the section, we will first introduce synaptic devices that can sense external stimuli: light (Li et al., 2016; Lee et al., 2017; Qian et al., 2018; Wang et al., 2018c,d; Ahmed et al., 2019), sound (He et al., 2019), chemicals (Giordani et al., 2017; Song et al., 2019), and PH (Liu N. et al., 2015; Liu et al., 2019). Such devices can convert the external stimuli to the electrical signals, which can play a monitoring role for human to avoid being hurt. Then the incorporation of synaptic and sensing devices is displayed. Finally, the artificial sensory neuron systems are shown.

### Synaptic Devices With Sensing Capabilities

**FIGURE 10:**
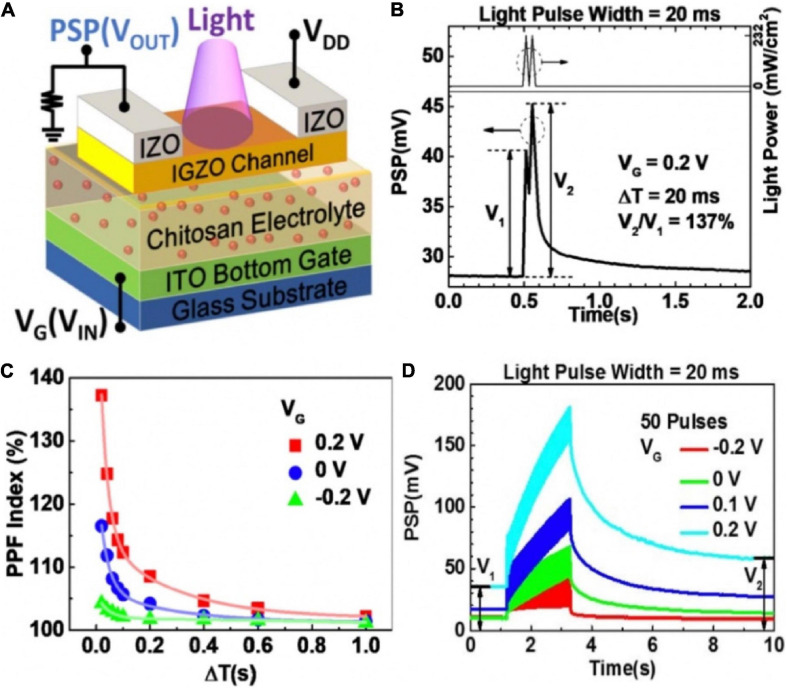
**(A)** Schematic diagram of the photonic neuromorphic transistor loaded with a resistor. **(B)** PPF realized in the synaptic transistor with light input pulses. **(C)** PPF index decreases with increased ΔT under various V_*G*_. **(D)** EPSPs triggered by a series of 50 light pulses under different V_*G*_ (adapted from Yang Y. et al., 2018).

The employ of light-sensitive materials in neuromorphic devices is favorable for artificial sensory neuron, because photonic synapses have the merits of large bandwidths and no electrical energy loss at interconnections (Sun et al., 2018; Dai et al., 2019; Wang et al., 2019). Yang Y. et al. (2018) proposed an IGZO-based synaptic transistor in series with a 10 MΩ resistor to realize photo-sensing application (Figure 10A). The light pulse, the output potential (V_*OUT*_) and conductance of the channel were regarded as input, post-synaptic potential (PSP) and synaptic weight, respectively. When incident light illuminated the IGZO channel layer, the channel conductance was reduced, and photocurrent could be generated. Two successive light spikes (405 nm, 232 mW cm^–2^, 20 ms) were applied on the channel, PPF characteristic was mimicked (Figure 10B). The PPF index gradually decreased with the increasing inter-spike interval (Figure 10C). Figure 10D displayed EPSP triggered by light pulse could be modulated by gate voltage. Besides, depression to potentiation mode transition was also displayed by gate voltage modulation.

**FIGURE 11:**
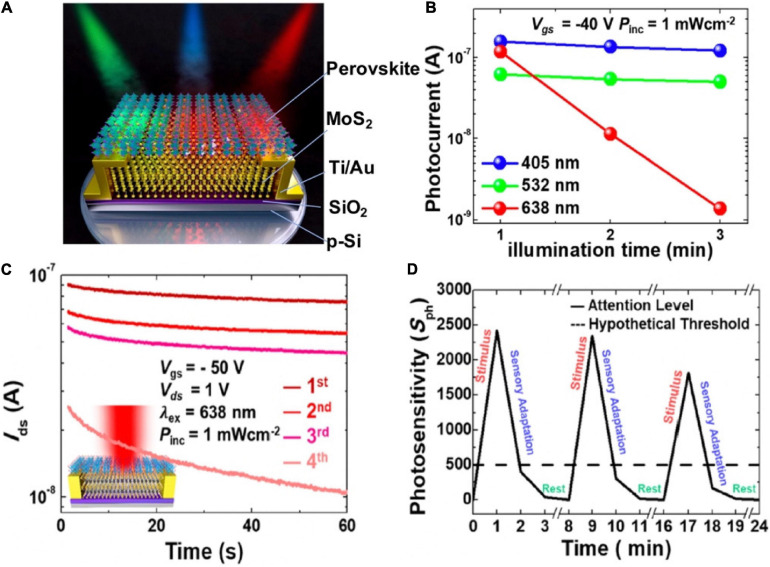
**(A)** Phototransistor under illumination of blue, green, and red light. **(B)** Variation trend of photocurrent in the phototransistor under RGB light illumination time. **(C)** Distinction of the photocurrents in the phototransistor under constant red light illumination with respect to time in four measurement. **(D)** Photosensitivity of the device during emulating sensory adaptation (adapted from Hong et al., 2020).

Except for artificial synaptic functions, biological behaviors at sensory receptors are highly demanded on synaptic devices. A synaptic phototransistor based on the hybrid structure of transition-metal dichalcogenide (TMD) and mixed halide perovskite [CsPb(Br_0.5_I_0.5_)_3_] emulated the human sensory adaptation to constant light (Figure 11A; Hong et al., 2020). The lessening in the sensitivity of sensory system toward a constant stimulus over time was called sensory adaptation. The combination of TMDs and perovskite overcame the weak light absorption of TMDs, because the photoexcited charges transferred from the perovskite to the MoS_2_ channel via the differences in band edge. As time went by, the photocurrent of the synaptic device under red light illumination degraded drastically within 3 min (Figure 11B). The phenomenon indicated the device could be applied for sensory-adaptation; the time-resolved photo-response of the device under continuous red-light illumination (Figure 11C) confirmed this. The synaptic device could also mimic the reversibility behavior of sensory-adaptation (Figure 11D), even though the recovery rate dropped slightly as the sequence repeated. The photosensory adaptation behavior of the device with selective light can be applied to intelligent sensors, and biomedical imaging.

**FIGURE 12:**
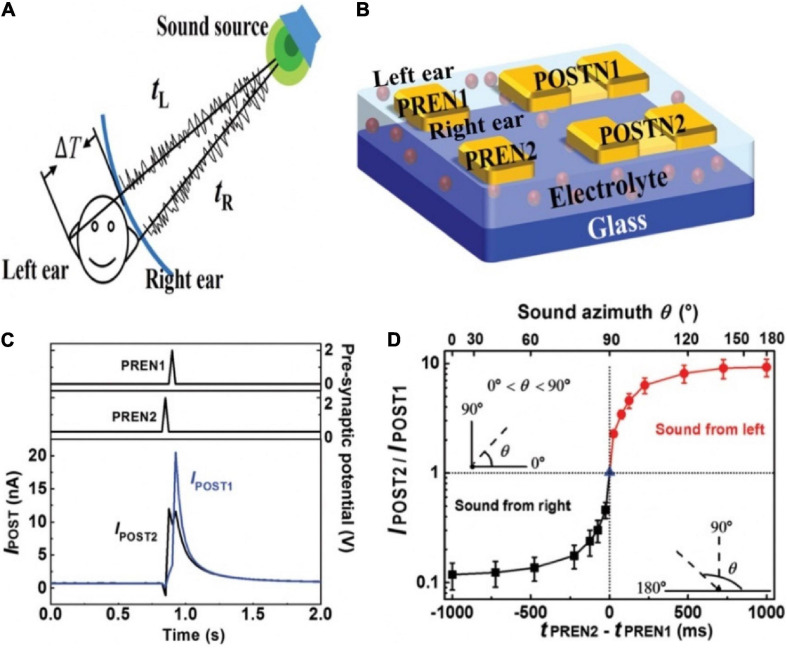
**(A)** Schematic diagram of sound location by binaural effect in human brain. **(B)** Sound location in artificial neural network based on the transistor. **(C)** Postsynaptic currents of POSTN2 and POSTN1 when sound comes from right direction. **(D)** I_*POST2*_/I_*POST1*_ changes with time interval and sound azimuth (adapted from He et al., 2019).

The sound location function of human brain was mimicked by artificial neural network based on a multi-terminal IGZO neuromorphic transistor (He et al., 2019). The schematic diagram of locating sound was shown in the Figure 12A. Sound azimuth detecting function was realized by a neural system that was consisted of two gate electrodes (PREN1 and PREN2) and two pairs of S/D terminals (POSTN1 and POSTN2). PREN1 and PREN2 were regarded as the left and right ears, respectively, and were fully connected to POSTN1 and POSTN2 (Figure 12B). The connection strength between PREN and two POSTNs were diagonal, implying that one POSTN processed spikes transmitted by the synapses in a strong/weak order while the other processed information in the opposite sequence. When the sound was from the right direction, POSTN1 first processed the signal transmitted by a weak synapse and then processed the signal transmitted by a strong synapse. While POSTN2 processed information in the opposite. Consequently, the amplitude of I_*POST1*_ was larger than that I_*POST2*_ (Figure 12C). The ratio of the amplitude of I_*POST2*_/I_*POST1*_ as the function of (t_*PREN2*_ − t_*PREN1*_) and the sound azimuth was shown in Figure 12D. This time-dependent recognition shows the potential that the artificial neural network can detect the sound azimuth.

**FIGURE 13:**
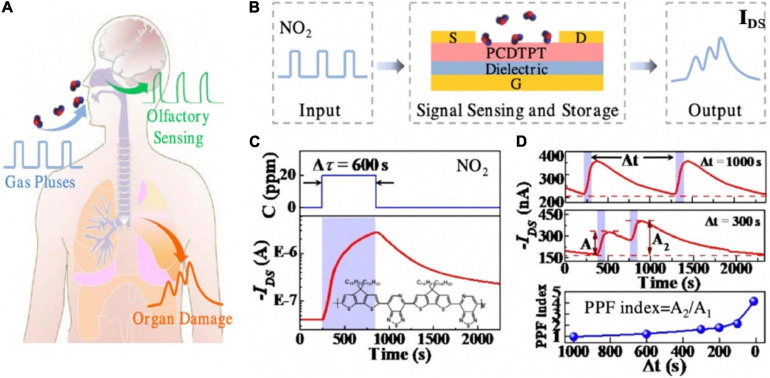
**(A)** Schematic image that shows different injuries of hazardous gas on human and organs and olfactory. **(B)** Schematic of the operation concept of the artificial organ-damage device. **(C)** Typical response/recovery characteristics of the device to 20 ppm NO_2_. **(D)** Real-time I_*DS*_ to two successive 20 ppm NO_2_ pulses at time interval 1,000 and 300 s, respectively, and the PPF index as the function of pulse interval (adapted from Song et al., 2019).

An organic transistor which had the function of detecting hazardous gas (NO_2_) leakage paved the way of human health monitoring (Song et al., 2019). As depicted in the Figure 13A, human organs would be damaged under the influence of toxic gas. A large number of studies have shown that prolonged exposure to toxic gas environments could have varying degrees of health effects: respiratory diseases and even lung cancer (Ezratty et al., 2014; Hettfleisch et al., 2017; Mentz et al., 2018). The flexible toxic gas detection device was fabricated with PVA dielectric, PCDTPT channel, gold back-gate and source/drain electrodes (Figure 13B). Hazardous gas acted as the input pulse, PCDTPT channel layer was used as the gas sensing and storage layer. NO_2_ molecules had the property of withdrawing electron. When there was 20 ppm NO_2_, the captured NO_2_ remained on the surface of the channel, acting as electron trapping centers, contributed to the increase of holes in the channel, and resulted in the mounting of I_*ds*_. After removing the pulse for 600 s, I_*ds*_ had a greatly slow decay process, and did not restore to the original value (Figure 13C). That was the reason of the absence of external energy such as thermal/photo energy at room temperature. The PPF was demonstrated in Figure 13D. The functions implemented by the device provide great potential for human health monitoring and non-invasive diagnosis.

**FIGURE 14:**
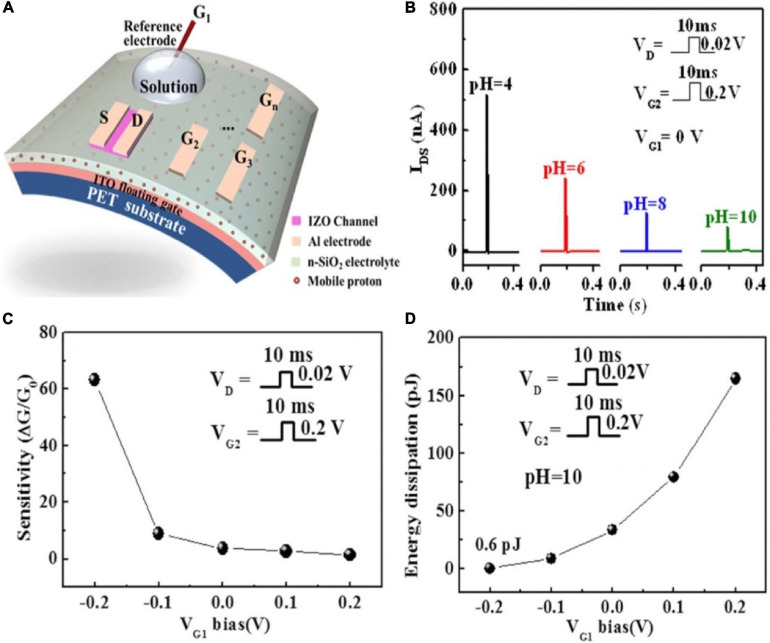
**(A)** Schematic of the pH-sensing IZO-based synaptic transistor. **(B)** The change of current with pH value. **(C)** The change of sensitivity with V_*G1*_ bias (pH = 10). **(D)** The change of energy dissipation with V_*G1*_ bias (pH = 10) (adapted from Liu N. et al., 2015).

As shown in Figure 14A, a pH-sensing IZO-based synaptic transistor with multiple gate electrode was demonstrated. An Ag/AgCl reference electrode immersed into a pH buffer solution droplet acted as the sensing gate (G_1_). In-plane Al gate electrodes were used as control gates (Liu N. et al., 2015). The IZO channel could be efficiently tuned by the sensing and control gates, because of the electric field that was coupled by sensing and control gates. As the pH increased, the magnitude of EPSC decreased (Figure 14B). Since the hydrogen ions in the solution would generate a charge repulsion reaction with the protons at the electrolyte interface, and more protons accumulated in the area below the channel, the EPSC was relatively increased. It could be inferred that an appropriate negative bias applied on the sensing gate would increase the sensitivity of the device (Figure 14C) and lessen the energy dissipation (Figure 14D).

### Artificial Synapses Combined With Sensors

The achievable functions of sensory synaptic devices under limited material selection are relatively simple and cannot show diverse synaptic characteristics. Hence, higher level of device structures needs to be designed to make up for this shortcoming (Chen et al., 2018; Seo et al., 2018; Wang et al., 2018a). By integrating with various sensors, the external stimuli can be utilized as the inputs of synaptic devices to realize the emulation of more complex biological functions. The combination of the two parts promotes the development of neuromorphic engineering in the direction of sensory applications.

**FIGURE 15:**
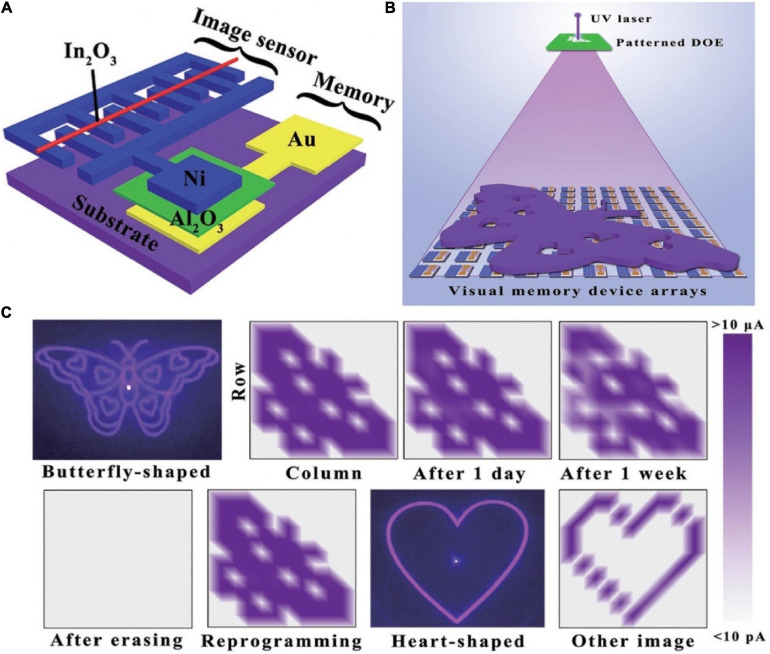
**(A)** Schematic diagram of the bio-inspired visual memory unit integrated by resistive switching memory device and image sensor. **(B)** Schematic diagram of detecting and memorizing the light distribution which generated from patterned diffraction optical element (DOE). **(C)** Schematic of the resistance states between memory device and image sensor (adapted from Chen et al., 2018).

Human visual system is essential for the knowledge acquisition, which via eyes to sense light and brain to storage image information. Herein, structure of the device that integrated image sensor and memory to emulate visual memory was shown in the Figure 15A (Chen et al., 2018). In_2_O_3_ was chosen for the functional material to detect UV light, for it was light-sensitive; Al_2_O_3_ was exploited as the memory material on account of the excellent bipolar resistive switching feature. When In_2_O_3_ was irradiated with UV light, the charge carrier concentration in In_2_O_3_ increased. So, resistance state of the image sensor was transformed from high resistance to low resistance, due to the connection with the image sensor, the resistance switching state of the memory from OFF to ON. Even after the UV was removed, the state of the memory remained until the application of the reset potential, implying that the lighting information was stored in the memory. Visual memory arrays composed of 10 × 10 pixels were fabricated to exhibit imaging and memorizing a butterfly-like pattern (Figure 15B), each pixel was formed by image sensor and memory to compose a visual unit. Only the pixels exposed to the patterned UV light worked normally, and ultimately form the targeted pattern which could retain for 1 week (Figure 15C).

Wang and co-workers presented a light-triggered organic neuromorphic device (LOND) to emulate the retinal functionalities (Wang et al., 2018a). A flexible array was composed of 5 × 6 pixels then were curved into hemispheric surface to imitate the retinal functions. The NIR and green light with the identical frequency (64 Hz) and intensity (10.80 mW cm^–2^) were shed on the array. In contrast, green light evoked a higher current level, even after 1,800 s, still had 65% of original signals. As mentioned above, the LONDs could achieve the goal of wavelength-recognition, that was expressed by the degree of non-volatility.

Tactile sense is indispensable for normal human activities. It has the functions of protecting the human body from injury and diagnosing diseases. In recent years, there have been an amount of studies on emulating haptic function. Yet the single function imitations of synaptic devices lack the functional memory of tactile, such devices are awkward when reacting to the same motion (Tabot et al., 2013; Foley, 2016). Therefore, the development of tactile-sensing system is of great significance for prosthetics and robotics (Zang et al., 2017; Kim et al., 2018; Zhang C. et al., 2019; Zhang et al., 2020).

**FIGURE 16:**
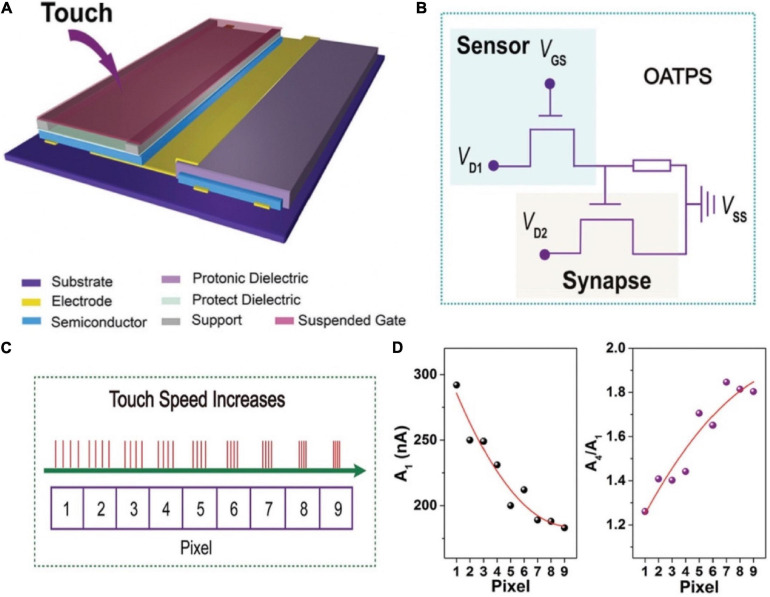
**(A)** Schematic illustration of the DOT-TPS. **(B)** Equivalent electrical circuit of the DOT-TPS. **(C)** Four continuous and repeated touch cycles applied to the nine pixel. **(D)** The EPSC (A_1_) and the gain A_4_/A_1_ for each pixel (adapted from Zang et al., 2017).

In 2016, Zang et al. (2017) demonstrated a dual-organic-transistor (DOT) based tactile perception element (TPE) to achieve the goal that dynamic signal transduction and information processing work in a device. A suspended-gate organic field-effect transistor was employed as the pressure sensing element, and integrated with a synaptic device, forming a prototype DOT-TPE (Figure 16A). The equivalent electrical circuit of the DOT-TPE was exhibited in Figure 16B. When there was an external pressure on the sensor, causing a change in capacitance of the dielectric. Consequently, the conductivity of the sensor was tuned, resulting in the transport of presynaptic spike to the synaptic transistor. Tactile information was collected by monitoring the EPSC of synaptic transistor. A 3 × 3 pixels DOT-TPE array was constructed to emulated the perception of dynamic mechanical pressure, and four bias cycles of retentive pressure at mounting frequencies were applied on pixel 1–9 (Figure 16C). Figure 16D showed a falling EPSC of the first press action (A1) from pixel 1–9, because the increased frequency made the contact time shorter. To the opposite, the gain A4/A1 of every pixel grew from 1.2 to 1.8, indicating the high-pass temporal filtering feature of the tactile-perception system.

**FIGURE 17:**
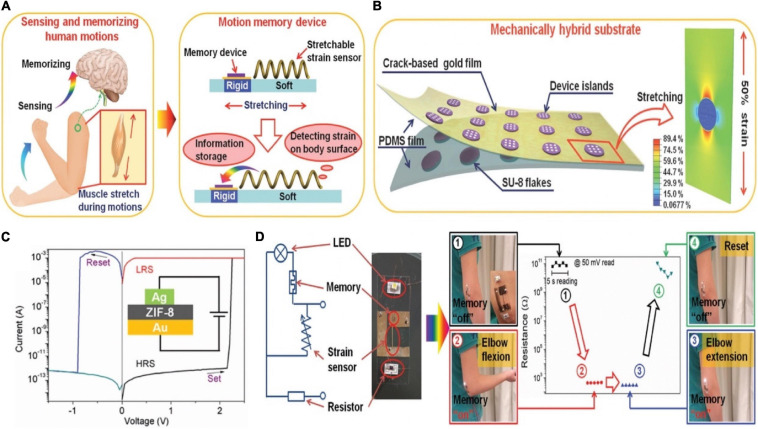
**(A)** Explanation of the biological concept of motions (left) and the corresponding bionic motion memory device (right). **(B)** Schematic diagram of the specific structure of the device. **(C)** I–V curve of the Ag/ZIF-8/Au device (insert: structure of the device). **(D)** The equivalent electrical circuit of the motion memory system (left), and the detecting and storing information function of the system on elbow flexion (right) (adapted from Liu et al., 2017).

The movement of human limbs benefits from the movement of muscles contracting and pulling bones to produce joints, and also requires the adjustment of the nervous system (Graziano, 2006; Urgesi et al., 2006; Stefan et al., 2008). Integrating motion sensor and memory had great significance for robotics and health monitoring systems (Figure 17A; Liu et al., 2017). A hybrid substrate that was spatially separated into different mechanical properties was introduced. Therefore, both fragile memory device and flexible sensor could be combined as a uniform unit (Figure 17B). The memory was based on ZIF-8, and gold film with microcrack morphology/Ag worked as the B/T electrode. It was worth noting that gold film with microcrack morphology had a good ductility under stretching (Lacour et al., 2003; Graudejus et al., 2012; Liu et al., 2014). The I-V characteristic curves of the memory showed typical resistance switching property, which implied its non-volatile feature (Figure 17C). For the purpose of monitoring and storing the information of the elbow telescopic state, a simple circuit was built as shown in Figure 17D. The strain sensor and memory device were fabricated on the mechanical hybrid substrate, and the LED connected with the memory was adopted to observe the state of HRS and LRS. The circuit chip was attached onto the arm near the elbow. When elbow was in the extension state, the telescopic state of the sensor had not changed, i.e., the conductance remained original, so the memory showed HRS and the LED was dark. Correspondingly, the flexing of elbow caused the elongation of the sensor, which switched the memory to the “on” state, and lighted the LED until a reset voltage was applied.

### Integration for Constructing Artificial Neural Systems

The previous section introduced the combination of sensors and synaptic devices to achieve more complex functionalities. Nevertheless, the postsynaptic signals of the synaptic devices have not been employed to display practical functions such as motor behavior, distinguishing and identifying tasks. This section will introduce artificial neurons that are constructed by sensors, synaptic devices and proper electric elements. Such artificial neurons can utilize their postsynaptic signals to achieve some sophisticated bionics functions. The artificial neural systems play the key role in the field of artificial intelligence to mimic the “cognitive” function of human (Langley, 2011; Jordan and Mitchell, 2015; Zhang C. et al., 2019).

**FIGURE 18:**
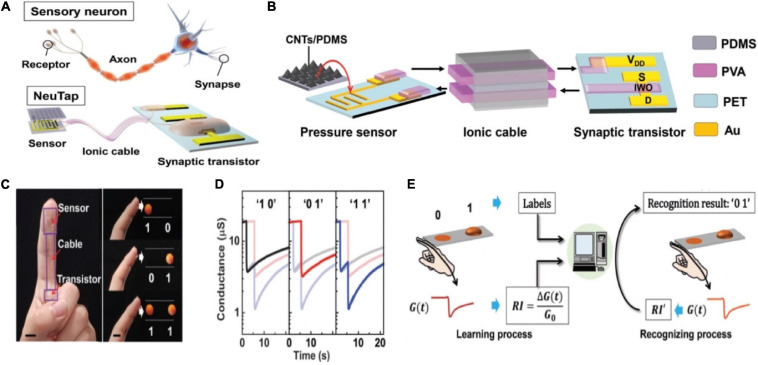
**(A)** Sensory neuron compared to the NeuTap. **(B)** Schematic of the details of the NeuTap. **(C)** Digital image of the NeuTap on one finger and the pattern pairs as well as the corresponding two-bit binary code labels. **(D)** The responses of the NeuTap to three pattern pairs. **(E)** The machine learning method for the perceptual learning emulation (adapted from Wan et al., 2018).

A neuromorphic tactile processing (NeuTap) system composed of resistive pressure sensor, ductile ionic cable and synaptic transistor was exerted to mimic the sensory neuron (Figures 18A,B; Wan et al., 2018). The applied voltage on V_*DD*_ generated a voltage drop on PVA wires/pressure sensor/semiconducting channel. Once pressure was applied to the sensor, the resistance decreased rapidly to produce a voltage drop via the PVA wires, that was equivalent to applying a voltage on the PVA wires. The voltage created an EDL at the IWO/PVA interface, tuning the IWO channel conductance. A NeuTap with two sensing terminals was fabricated to mimic the integrated functions for spatiotemporal correlated sensory stimulation. As a proof-of-concept, tactile pattern recognition was completed via one sensing terminal in the NeuTap neuron, the neuron was attached to a finger for the experiment. Two patterns in a row were used for recognition; the convex pattern was marked as “1” and the flat patterns was as “0,” so that each set of patterns could be labeled as “00,” “01,” “10,” and “11” in the form of binary code (Figure 18C). The typical current responses of the NeuTap with three various pattern pairs were depicted in Figure 18D. Supervised learning method was employed in NeuTap to imitate the perceptual learning process. The change in channel conductance was defined as the recognition index (RI) (Figure 18E). RI data and their corresponding labels were employed as the training data, which were imported into the computer program to partition the boundaries for each pattern. The unlabeled RI data were inputted into the computer, then the computer compared the values with “learned” boundaries to infer the labels of the patterns. The 44% error rate could be decreased to 0.4% after six learning times, that resembled the perceptual learning process.

**FIGURE 19:**
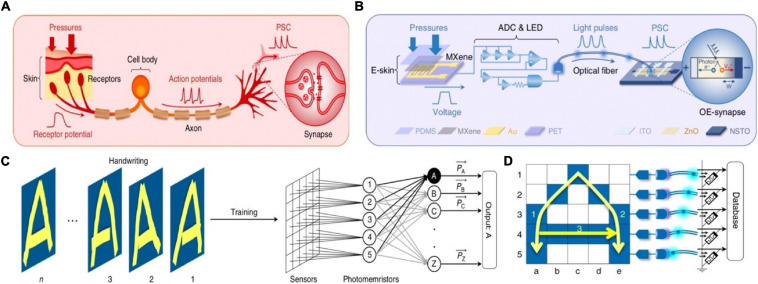
**(A)** The biological afferent nerve. **(B)** The corresponding artificial afferent nerve. **(C)** Schematic of handwriting recognition with trait extraction and learning. **(D)** structure and working principle of the artificial neural network (adapted from Tan et al., 2020).

An optoelectronic spiking sensory neuron system with pressure sensing, perceptual learning and memory ability has been reported recently (Tan et al., 2020). The system was operated by MXene-based flexible receptors, analogy to E-skin, to detect the external pressure and convert it to voltage signal. Subsequently, the signal was transmitted to the (analog-to-digital) ADC&LED section, and finally integrated into the synaptic photo-memristors in the form of optical spikes (Figures 19A,B). The reason of the receptors and the synaptic photo-memristors were connected in the form of optical was that spike coding was more robust than voltage coding (Burke and Ivory, 2008; Kim et al., 2018). A 5 × 5 sensors array was connected with an ADC-LED and a synaptic photo-memristor in order to recognize handwriting through training (Figure 19C). To simplify the handling of information, instead of processing the 25 data streams, the spiking proportions(P), defined as P = t_*spiking*_/t_*writing*_, of the five synaptic photo-memristors was extracted as a five-dimensional (5D) feature for the recognition and learning processes. The PSCs of the five photo-memristors corresponding to the input of the alphabet (Figure 19D). The recognition accuracy was improved from ∼58% after the first training cycle to 84% after 10 training cycles. The successful implementations of the functions in the above work provide great examples of brain-like learning and promote the development of artificial intelligence.

**FIGURE 20:**
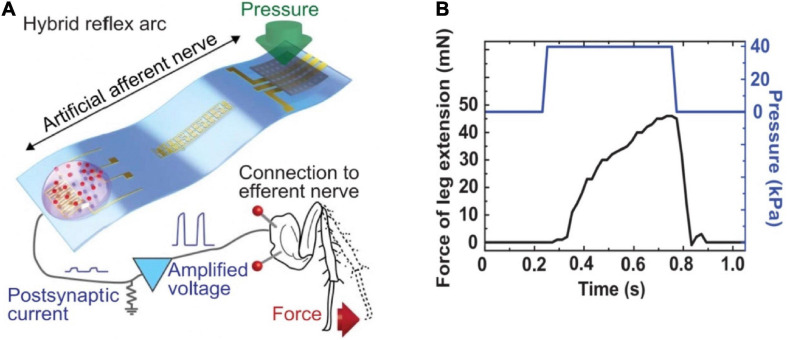
**(A)** Schematic diagram of the hybrid reflex. **(B)** Force measured from the tibial extensor muscle when pressure is applied on the hybrid reflex arc (adapted from Kim et al., 2018).

An artificial afferent nerve based on flexible organic electronics was demonstrated and used to emulate the function of slowly adapting type I (SA-I) sensory neurons (Pruszynski and Johansson, 2014; Kim et al., 2018). The artificial afferent nerve achieved the imitation of biological afferent nerves by the components of resistive pressure sensors/organic ring oscillators/a synaptic transistor corresponding to the receptor, nerve fiber, biological synapse, respectively. The pressure sensors were connected to a ring oscillator to convert external tactile stimulation into voltage pulses, then the electrical signals were integrated and transformed to the synaptic transistor by multiple electrodes. Ultimately, by the process above, the pressure stimuli were output in the form of postsynaptic currents. The highlight of this work was that a discoid cockroach was connected with the artificial afferent nerve to constitute a monosynaptic reflex arc (Figure 20A). The applied pressure information was collected, converted by the artificial afferent nerve, and finally outputted to a detached cockroach leg in the form of post-synaptic current. The post-synaptic current would actuate the tibial extensor muscle of cockroach leg (Figure 20B). The successful application of the artificial afferent nerve on the legs of cockroaches is of great significance for prosthetics and neurorobotics, and sets a solid foundation for the construction of neural networks.

**FIGURE 21:**
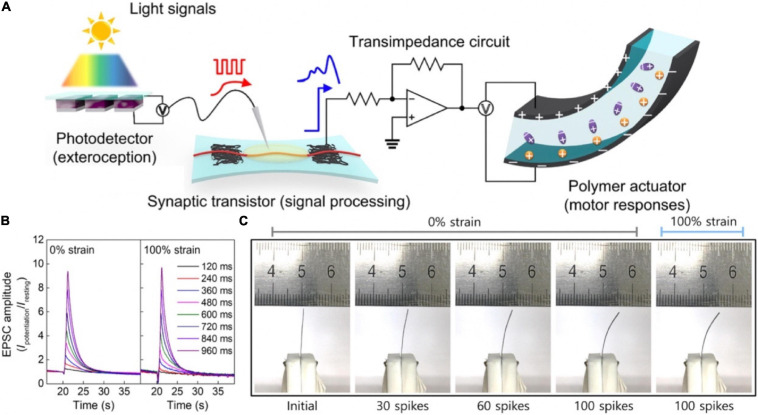
**(A)** The structure diagram of artificial sensorimotor nervous system. **(B)** SDDP from 120 to 960 ms under 0 and 100% strains. **(C)** Digital images of polymer actuator controlled by various spikes with 0 or 100% strain (adapted from Lee et al., 2018).

Similarly, an artificial sensorimotor nervous system was constructed (Figure 21A; Lee et al., 2018). In the system, photodetector converted optical signals to voltage spikes that were applied to a synaptic transistor as the presynaptic input signals to trigger EPSCs, and a polymer actuator was connected with the above synaptic transistor via a transimpedance circuit; the polymer actuator was controlled by the EPSCs of the synaptic transistor (Figure 21C). Therefore, the artificial nervous system could achieve biomimetic natural motion. The electrical characteristics of the stretchable synaptic transistor under 0 and 100% strains were almost the same (Figure 21B), indicating the transistor was stable even under strain. The sensorimotor synaptic system would promote the development of next-generation bioinspired soft electronics, neural prostheses, and neurologically inspired robotics.

## Summary and Perspectives

In this review, we have introduced various neuromorphic devices based on various materials, such as electrochemical metallized materials, phase-change materials, ferroelectric materials and ionic/electronic hybrid materials. 2-terminal devices have the merits of simple structure, small physical size, and easily to be integrated on a large scale. three-terminal (3-T) devices have the merits of relatively controllable test parameters, clear operating mechanism. Through appropriate material optimization and structural design, 3-T neuromorphic transistors can convert external stimuli (light, pressure, PH, etc.) into electrical signals efficiently. Multi-terminal neuromorphic transistors can spatiotemporally integrate input signals from multi-presynaptic terminals and facilitate synergistical sensing and perception.

For mimicking the functions of neural perception, neuromorphic devices that can sense the external environment accurately (light, sound, chemical, PH) are essential. Afterward, in order to meet the sensing and learning capabilities of neural systems, the integration of sensory and neuromorphic devices is particularly necessary, by which can make the systems sense external stimulus and store the relevant information. Such systems finally can be employed in humanoid robotic applications. What’s more, the monosynaptic reflex arc assembled by sensory and motor neuron has the potential to replace damaged nerves, and being used as a tackle in neuroscience for studying sensory and motor neuron diseases.

The development of neuromorphic devices for bionic sensing and perception applications has greatly promoted the field of artificial intelligence. First of all, there are still limitations in the selection of materials that respond to external stimuli (PH, sound, chemical, touch). For instance, to monitor toxic chemical, the device must employ materials that react specifically with the target. Secondly, for the construction of the artificial neural systems, there is still huge research space for structural design and optimization. Take pressure sensory neuron as an example, which is composed of three parts: pressure receptor, axon, and synapse. Lots of efforts are needed in the connection between the receptor and the synapse, that is, the afferent nerve part: the conductivity, transmission efficiency and the mechanical flexibility of the connection material must be considered, and the impact of the interference generated by the bionic sensor on the performance of the neuromorphic device needs to be further minimized. The normal maintenance of mammals and even the human body is inseparable from complex physiological adjustments. Insufficient understanding of the mechanism of the biosensing systems is also a major challenge that limits the bionic sensing and perception application of neuromorphic devices. So more interdisciplinary cooperation is needed in the area of neuromorphic devices and bionic sensing and perception application.

## Author Contributions

QW and MZ conceptualized the concept and wrote the manuscript. YH and CZ participated in writing the manuscript. All authors contributed to the article and approved the submitted version.

## Conflict of Interest

The authors declare that the research was conducted in the absence of any commercial or financial relationships that could be construed as a potential conflict of interest.
